# Impact of Neoadjuvant Chemotherapy on Skeletal Muscle Mass in Colorectal Cancer Patients Undergoing Curative Surgical Resection: A Single-Centre Retrospective Observational Study

**DOI:** 10.7759/cureus.89389

**Published:** 2025-08-05

**Authors:** Mahmud Riad, Mohamed Hassan, Kalyani Nair, Amr K Ebrahim, Hatim Albirnawi, Henry Vowles, Serena Merchant, Moneet Gill, Dinesh Balasubramaniam

**Affiliations:** 1 Colorectal Surgery, Tunbridge Wells Hospital, Tunbridge Wells, GBR; 2 General Surgery, Maidstone and Tunbridge Wells NHS Trust, Tunbridge Wells, GBR; 3 Surgery, Maidstone and Tunbridge Wells Hospital, Maidstone, GBR; 4 General Surgery, Cairo University, Cairo, EGY; 5 Upper GI Surgery, Maidstone and Tunbridge Wells NHS Trust, Maidstone, GBR; 6 Trauma and Orthopedics, Maidstone and Tunbridge Wells NHS Trust, Tunbridge Wells, GBR; 7 General Surgery, Maidstone and Tunbridge Wells NHS Trust, Maidstone, GBR

**Keywords:** colon cancer, neoadjuvant chemotherapy(nact), psoas muscle mass, psoas muscle mass index, skeletal muscle index

## Abstract

Background

Neoadjuvant chemotherapy is often given before surgery in colorectal cancer to improve tumour resectability. However, its effects on skeletal muscle mass, which may influence post-operative recovery and functional outcomes, remain unclear. This study evaluates the impact of neoadjuvant chemotherapy on skeletal muscle mass in colorectal cancer patients undergoing curative surgery.

Aim

The aim of this study is to assess the effect of neoadjuvant chemotherapy on skeletal muscle mass in patients with colorectal cancer undergoing curative surgical resection.

Methods

A total of 100 patients who underwent surgical treatment for colorectal cancer between 2017 and 2022 were randomised into two groups: a chemotherapy group (n=50) that received neoadjuvant chemotherapy and a control group (n=50) that underwent surgery alone. Skeletal muscle area (cm²) was measured using CT scans both pre- and post-operatively: rectus abdominis at L1 and psoas at L4. Values were normalised for patient height squared to derive the skeletal muscle index (cm²/m²). Post-operative complications were recorded.

Results

The study enrolled 100 patients (median age 66.7 years, IQR 13.46; 58% male). No significant reduction in rectus or psoas muscle mass was observed pre- and post-chemotherapy in the treatment group (rectus: 4.33±3.8 vs. 4.03±1.96, p=0.567; psoas: 8.85±1.97 vs. 8.91±1.96; p=0.745). Baseline rectus muscle mass was comparable between groups (4.33±3.8 vs. 4.8±2.06; p=0.444). Post-operatively, the chemotherapy group showed lower rectus and psoas muscle mass compared to controls (rectus: 3.77±1.36 vs. 4.52±1.66, p=0.016; psoas: 9.09±1.93 vs. 10.13±2.86; p=0.037). No significant differences in pre- and post-treatment muscle mass change between groups (p>0.05). The chemotherapy group reported a higher incidence of falls (12% vs. 0%, p=0.027) and post-operative muscle weakness (22% vs. 0%, p=0.0004).

Conclusion

Neoadjuvant chemotherapy did not significantly impact short-term muscle mass loss in colorectal cancer patients undergoing curative surgery. However, a higher incidence of post-operative functional decline was observed, warranting further investigation.

## Introduction

Colorectal cancer is one of the most common cancers worldwide. In 2022, more than 1.9 million cases were diagnosed, and it is the second most common cause of cancer death, leading to more than 900,000 deaths per year [1]. Neoadjuvant chemotherapy in colorectal cancer of curative intent is an important consideration in cancer treatment. It is a form of chemotherapy that is administered before the primary treatment, such as surgery, to reduce the size of the tumour, treat micro-metastatic disease early, decrease local disease burden (potentially leading to more effective resections), improve treatment tolerability, and improve the chances of successful curative treatment [2].

There are several complications from chemotherapy. It can also have various effects on muscle mass, and these effects can vary depending on the type of chemotherapy drugs used, the duration of treatment, and the individual patient's response. Some common ways in which chemotherapy can impact muscle mass include skeletal muscle depletion (sarcopenia), an emerging issue where its closely related worse outcomes include limited physical abilities and high mortality [3]. However, nutritional status and changes in body composition have been shown to affect perioperative surgical outcomes such as length of hospital stay and complication rates [4].

Research on the impact of neoadjuvant chemotherapy on muscle mass in colorectal cancer patients is essential because chemotherapy can have both beneficial and adverse effects on the body. Chemotherapy drugs can potentially affect muscle mass and body composition, which, in turn, can have implications for a patient's overall health and recovery. However, these effects can vary from person to person. Strategies to mitigate these effects may include nutritional support, physical therapy, and exercise programmes tailored to the individual's needs.

This pilot study aimed to investigate the impact of neoadjuvant chemotherapy on muscle mass for patients with colorectal cancer with curative intent.

## Materials and methods

Study population and setting

Patients with curative intent surgical treatment of colorectal cancers between 2017 and 2022 were retrospectively recruited at an NHS district hospital, where they were randomised into two groups: a neoadjuvant chemotherapy group (n=50), who received neoadjuvant chemotherapy followed by surgery, and a control group (n=50), who underwent surgical treatment only. Both groups were matched for age and cancer stage.

The cross-sectional muscle area (cm²) was measured by computed tomography images, with rectus abdominis muscles measured at the L1 level and psoas muscles measured at the L4 level. This was normalised by the square of the height to obtain the skeletal muscle mass index (SMI) (cm²/m²). Post-operative events were recorded.

**Figure 1:**
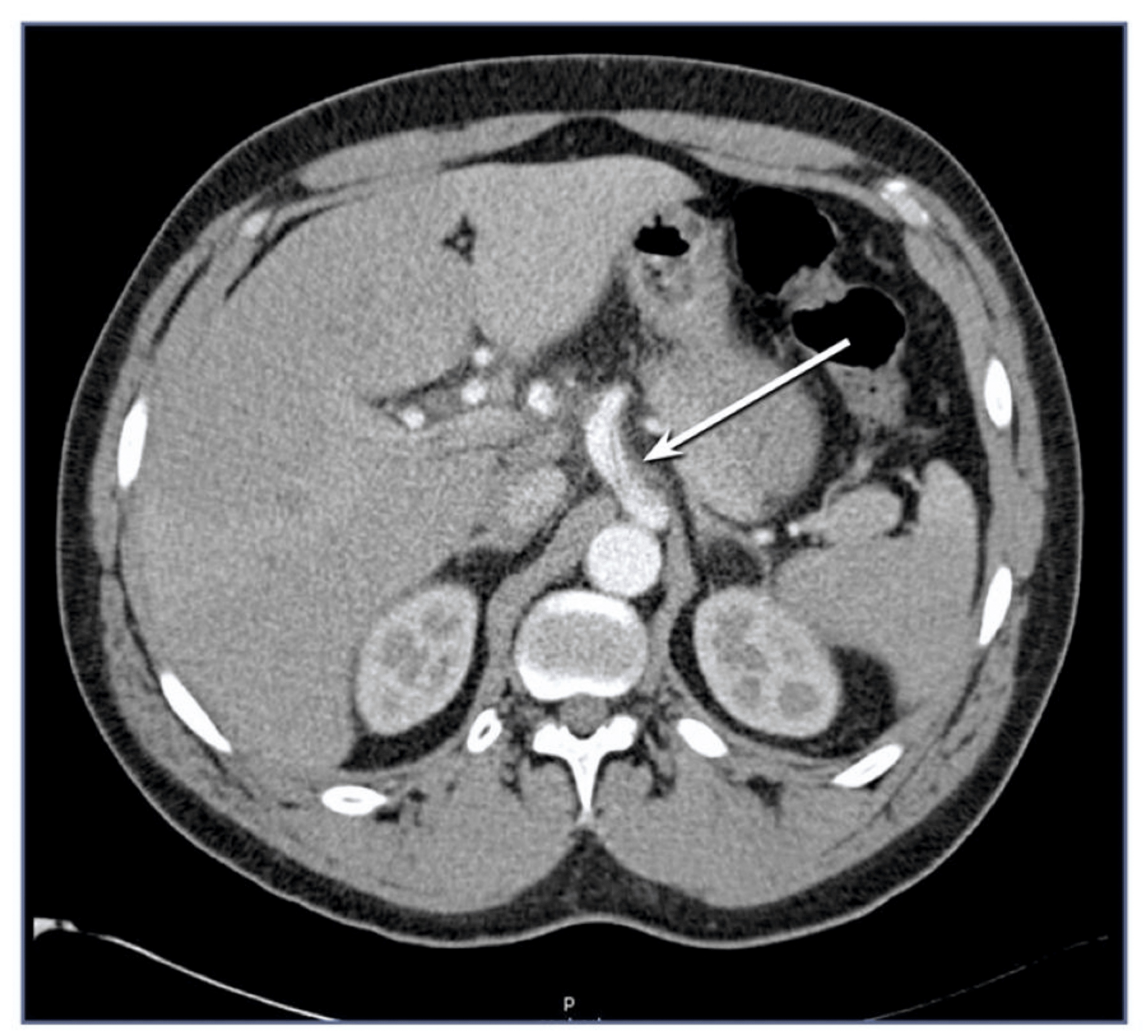
CT of the abdomen in the axial view shows the celiac trunk = level of L1 (white arrow).

**Figure 2:**
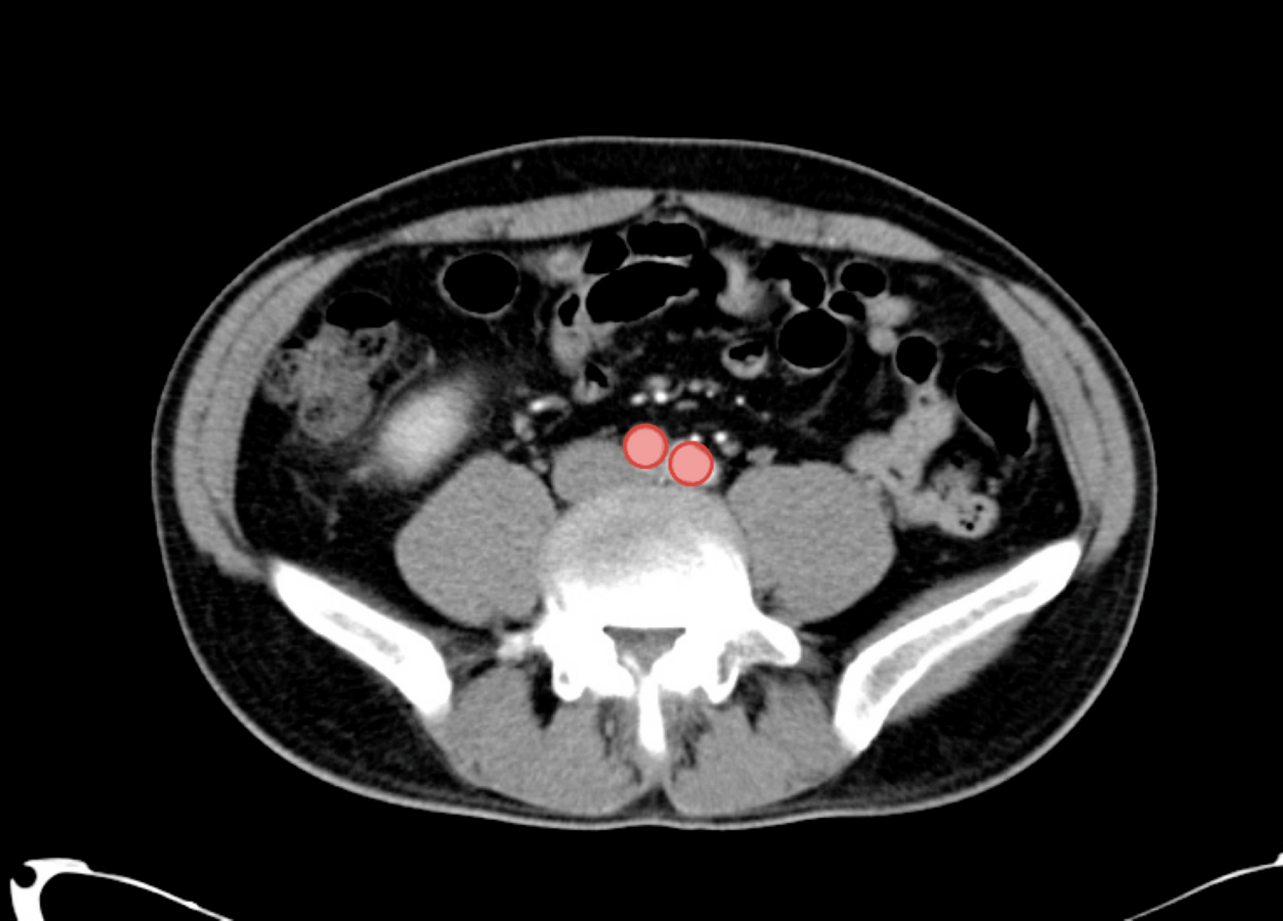
CT scan in the axial view showing the bifurcation of the aorta = level of L4 (red circles).

CT scans were performed at three time points: before the start of neoadjuvant chemotherapy (before performing surgery in the control group), after neoadjuvant chemotherapy, and after surgery. Skeletal muscle mass was measured using CT axial views at two anatomical landmarks: the rectus abdominis at the level of the celiac trunk (L1 vertebra, Figure 1) and the psoas muscles at the level of aortic bifurcation (L4 vertebra, Figure 2). Measurements were taken bilaterally in transverse and vertical dimensions using the Picture Archiving and Communication System (PACS). Measurements were performed by trained analysts.

Statistical analysis

Data were coded and entered using the Statistical Package for Social Science (SPSS) Version 26 (IBM Corp., Armonk, NY) and checked for normality using the Shapiro-Wilk test. Data were presented using numbers and percentages for qualitative variables, mean and standard deviation for quantitative normally distributed variables, and median and interquartile range for quantitative non-normally distributed variables. Comparison between groups was done using the chi-square test or Fisher’s exact test, where appropriate, for qualitative variables. Independent samples t-test was used for independent comparison of quantitative normally distributed variables, and paired samples t-test and repeated measurements ANOVA were used for dependent comparisons of quantitative normally distributed variables. For comparing quantitative non-normally distributed variables, a nonparametric Mann-Whitney test was used. A p-value of less than or equal to 0.05 was considered statistically significant.

## Results

Of the 100 patients, 50 received neoadjuvant chemotherapy prior to surgery and 50 underwent upfront surgical operations. Both groups of patients were matched for age and sex (Table 1).

**Table 1:** Baseline characteristics of patients (n=100) χ², chi-square test; U, Mann-Whitney test IQR, interquartile range

Variable	Neoadjuvant chemotherapy (n=50)	Control (n=50)	Test statistic	p-value
Sex (male/female)	28 (56%)/22 (44%)	30 (60%)/20 (40%)	χ² = 0.16	0.685
Age (median, IQR)	66.73 (58.8–72.2)	66.74 (58.4–72.6)	U = 1220.5	0.912

There was no statistically significant difference in rectus muscle mass between the chemotherapy and control groups at both the pre-surgical and post-chemotherapy stages (4.33±3.8 vs. 4.8±2.06, p=0.444 and 4.03±1.96 vs. 4.8±2.06, p=0.058, respectively) (Tables 2, 3).

**Table 2:** Pre-operative pre-chemotherapy skeletal muscle mass among patients (n=100) t, independent samples t-test

Muscle	Chemo (mean ± SD)	Control (mean ± SD)	t-value	p-value
Rectus muscles	4.33 ± 3.8	4.8 ± 2.06	-0.77	0.444
Psoas muscles	8.85 ± 1.97	10.21 ± 2.3	-3.19	0.002

**Table 3:** Pre-operative post-chemotherapy skeletal muscle mass among patients (n=100) t, independent samples t-test

Muscles	Chemotherapy (mean ± SD)	Control (mean ± SD)	t-value	p-value
Rectus muscles	4.03 ± 1.96	4.8 ± 2.06	-1.93	0.058
Psoas muscles	8.91 ± 1.96	10.21 ± 2.3	-3.07	0.003

Among the chemotherapy group, both rectus and psoas muscle mass did not significantly decrease before and after receiving chemotherapy (4.33±3.8 vs. 4.03±1.96, p=0.567 and 8.85±1.97 vs. 8.91±1.96, p=0.745, respectively) (Table 4).

**Table 4:** Changes in skeletal muscle mass before and after chemotherapy in the neoadjuvant group (n=50) t, paired samples t-test

Muscle group	Pre-chemotherapy (mean ± SD)	Post-chemotherapy (mean ± SD)	t-value	p-value
Rectus muscles	4.33 ± 3.8	4.03 ± 1.96	0.58	0.567
Psoas muscles	8.85 ± 1.97	8.91 ± 1.96	-0.33	0.745

Post-operative muscle mass was lower among the chemotherapy group than the control group for both rectus and psoas muscles (3.77±1.36 vs. 4.52±1.66, p=0.016 and 9.09±1.93 vs. 10.13±2.86, p=0.037, respectively) (Table 5).

**Table 5:** Post-operative skeletal muscle mass among patients (n=100) t, independent samples t-test

Muscles	Chemotherapy (mean ± SD)	Control (mean ± SD)	t-value	p-value
Rectus muscles	3.77 ± 1.36	4.52 ± 1.66	-2.45	0.016
Psoas muscles	9.09 ± 1.93	10.13 ± 2.86	-2.13	0.037

We compared changes in muscle mass within each group by calculating the difference between post-chemotherapy/pre-surgical and post-operative measurements. These changes were not statistically different between the groups (p>0.05) (Table 6).

**Table 6:** Percent change in skeletal muscle mass postoperatively compared to pre-surgical (post-chemotherapy) measurements (n=100) Values are expressed as median (IQR). U, Mann-Whitney U test

Muscles	Neoadjuvant chemotherapy (n=50)	Control (n=50)	U-value	p-value
Rectus muscles	-2.9 (-21.13, 21.23)	2.7 (-21.26, 15.32)	1,238	0.918
Psoas muscles	1.5 (-7.8, 7.65)	2.1 (-6.7, 7.8)	1,197	0.669

The chemotherapy group reported more falls (12%, p=0.027) and more muscle weakness (22%, p=0.0004) in the post-operative period than the control group, which did not report any of these complications (Table 7).

**Table 7:** Post-operative events of patients (n=100) ¹Fisher’s exact test. ²Chi-square test value.

Complication	Neoadjuvant chemotherapy (n=50)	Control (n=50)	Test statistic	p-value
Chest infection	0 (0%)	3 (6%)	0.25¹	0.242
Anastomotic leak	0 (0%)	0 (0%)	–	–
Bowel obstruction	0 (0%)	2 (4%)	0.50¹	0.495
Reported falls	6 (12%)	0 (0%)	5.00²	0.027
Muscle weakness (subjective)	11 (22%)	0 (0%)	13.09²	0.0004
Burst abdomen	0 (0%)	0 (0%)	–	–
Superficial wound infection	8 (16%)	5 (10%)	0.80²	0.372
ITU stay >7 days	6 (12%)	2 (4%)	0.27¹	0.269

## Discussion

Low skeletal muscle mass is an emerging issue in oncology, where the amount of skeletal muscle mass loss varies widely across cancer types, with 5% to 89% of cancer patients having low skeletal muscle mass [5]. Low skeletal muscle mass at baseline has been reported to increase the incidence of disability among cancer patients and is associated with poor anti-tumour response. In addition, cancer patients with low skeletal muscle mass during cancer treatment have been reported to have a higher risk of mortality, cancer recurrence, and reduced quality of life [6].

In colorectal cancer patients, skeletal muscle area was used as a prognostic tool for sarcopenia [7]. In our study, we measured skeletal muscle mass index using longitudinal and transverse muscle diameters at L2, divided by square root of patients’ height; however, L3 skeletal muscle index (SMI) was used for screening pre-operative nutritional risk and diagnosing sarcopenia and considered as a potential clinical indicator that can be used to predict the prognosis of colorectal cancer patients [8]. In this study, in the chemotherapy group, muscle mass was expected to reduce after receiving chemotherapy when CT was repeated, but post-operative muscle mass was significantly lower in the chemotherapy group than the control group. However, other studies reported loss of muscle mass after chemotherapy. Griffin et al. [9] assessed total muscle and adipose tissue mass during adjuvant chemotherapy for pancreatic tumours that showed deterioration (diagnostic and post-chemotherapy CT skeletal muscle reduction; median, IQR 128.4, 32.7 vs. 120, 33.7, p<0.001). According to Guinan et al. [10], body composition analysis revealed a significant loss of SMI (mean [95% CI] loss 5.6 [3.7 to 7.5] cm^2^/m^2^, p < 0.001) for pre-operative chemoradiotherapy for oesophageal cancer.

Okuno et al. [11] showed SMI (mean± SD) pre-operative and post-operative chemotherapy (51.2±10.6 and 50.6±10.7, p=0.033) changes among colorectal patients with liver metastasis prior to surgical intervention, and Reisinger et al. [12] reported that the mean L3 index decreased significantly during neoadjuvant chemoradiotherapy for oesophageal cancer, which was measured in patients with both a pre- and post-chemoradiotherapy CT scan (from 50.9 ±8.5 cm^2^/m^2^ to 48.4 ±8.5 cm^2^/m^2^, p < 0.001).

Jang et al. [6] investigated skeletal muscle mass index pre-to post-chemotherapy and synthesised potential key factors, where mean difference in SMI during chemotherapy was 2.72 and that loss was 1.6 times higher among males than females, and Oflazoglu et al. [13] found that the incidence of sarcopenia increased with chemotherapy itself and with its duration in both non-metastatic and metastatic cancer patients. On the other hand, among patients with advanced lung cancer, many of them had an increase in muscle mass without receiving any additional cachexia therapy, and many of those patients were sarcopenic before starting chemotherapy. There was also a trend towards more gain in muscle mass among patients who had disease control following chemotherapy, suggesting that response to cancer treatment is fundamental for controlling cancer cachexia, as reported by Stene et al. [14].

Moreover, patients with resectable colorectal liver metastases receiving neoadjuvant chemotherapy had decreased skeletal muscle mass, and those with muscle loss >5% during neoadjuvant chemotherapy were less likely to undergo adjuvant chemotherapy than others [15]. Sarcopenia occurred in breast cancer patients when they began neoadjuvant chemotherapy, and the risk of muscle mass loss during treatment was alarmingly high, which necessitated a comprehensive evaluation of body composition [6].

In our study, the psoas muscle mass index did not show a significant reduction after chemotherapy, which should be further investigated and compared with results reported by Benedek et al. [16], where the low- and high-grade complication groups of colorectal cancer patients showed a significantly lower psoas muscle index.

Limitations

This study is limited by its retrospective design, small sample size, and single-centre setting, which may affect generalisability. Key confounding factors, such as nutritional status, comorbidities, physical activity, and performance status, were not assessed. Chemotherapy protocols were not uniform, and differences between regimens were not analysed due to limited numbers. Future multicentre, prospective studies are needed to validate these findings.

## Conclusions

Neoadjuvant chemotherapy does not cause significant short-term loss of skeletal muscle mass when assessed alone. However, patients undergoing both Neoadjuvant chemotherapy and surgery experience greater post-operative muscle depletion and functional impairment. Early identification of at-risk patients and perioperative interventions such as nutritional support and physiotherapy may mitigate these adverse effects.
